# Understanding the Awareness of Prenatal Genetic Screening Tests Among Pregnant Women in India: A Cross-Sectional Study

**DOI:** 10.7759/cureus.56932

**Published:** 2024-03-26

**Authors:** Sangeetha Arumugam, Sri Sowmya Kalluri, Vijayan Sharmila, Akarsh Mocherla, Nandha Kumar Subbiah, Jyoti P Kulkarni, Joy A Ghoshal

**Affiliations:** 1 Genetics Unit, Anatomy, All India Institute of Medical Sciences, Mangalagiri, IND; 2 Obstetrics and Gynecology, All India Institute of Medical Sciences, Mangalagiri, IND; 3 Obstetrics and Gynecology, Siddhartha Medical College, Government General Hospital, Vijayawada, IND

**Keywords:** down syndrome and other trisomies, chromosomal aneuploidies, nuchal translucency, prenatal care awareness, prenatal ultrasound, non-invasive prenatal test, prenatal maternal screening, prenatal genetic testing

## Abstract

Introduction

Genetic disorders pose a significant health challenge in India, with chromosomal abnormalities ranking second only to congenital anomalies in terms of disease burden. Prenatal testing offers a crucial strategy for identifying and managing these disorders. However, the awareness and understanding of prenatal screening tests among pregnant women in India remain understudied. This study aims to fill this gap by investigating the awareness quotient of prenatal screening tests for genetic disorders among pregnant women in India.

Methods

A hospital-based cross-sectional study was conducted at the Genetics Unit, Department of Anatomy, and Department of Obstetrics and Gynecology, All India Institute of Medical Sciences, Mangalagiri. Ethical clearance was obtained, and data were collected using a self-administered questionnaire covering demographic characteristics and awareness assessment. Descriptive statistics, chi-square tests, and logistic regression analysis were employed for data analysis.

Results

Among the 200 pregnant women surveyed, the majority demonstrated inadequate awareness of prenatal screening tests for genetic disorders, with only 36.5% possessing adequate knowledge. Significant associations were found between awareness levels and factors such as age, trimester of pregnancy, and education level. Notably, awareness about non-invasive prenatal testing (NIPT) was notably low at 7%, indicating a need for targeted educational interventions. Comparison with international studies revealed varying levels of awareness across different populations, highlighting the influence of socio-cultural factors and healthcare systems.

Conclusion

This study underscores the need for improved awareness of prenatal screening tests among pregnant women in India. Addressing disparities in awareness, particularly among younger age groups and those with lower education levels, is crucial for informed decision-making in prenatal care. Targeted educational interventions can empower pregnant women to make informed choices, ultimately contributing to better maternal and child health outcomes. Further research should explore the effectiveness of such interventions in diverse settings to enhance prenatal care delivery.

## Introduction

India reports a high prevalence of genetic disorders due to its vast population size. Chromosomal disorders rank second only to congenital anomalies in terms of disease burden. Each year, approximately 34000 infants with Down syndrome are born in India, marking the highest number globally [1]. Prenatal testing emerges as the most effective strategy for mitigating the impact of genetic disorders [2]. Traditionally, prenatal testing follows a protocol comprising screening and diagnostic tests. Screening tests, which are non-invasive, involve maternal serum screening (double/triple marker) combined with ultrasound scans, yielding an 80% detection rate [3]. The latest addition to screening tests is the non-invasive prenatal test (NIPT), providing a 99% detection rate by analyzing cell-free fetal DNA in maternal plasma [4,5]. Invasive prenatal diagnostic tests are reserved for cases where screening tests yield positive results.

The acceptance of prenatal screening tests among pregnant women depends upon their awareness of genetic disorders, potential fetal risks, cost, availability, accuracy, turnaround time, and benefits. While numerous studies have explored attitudes, awareness, and acceptance of prenatal testing in various populations and regions globally, there is a scarcity of literature addressing knowledge and acceptance among pregnant women in India [6-9].

Experts within India have advocated for a nationwide, government-funded program aimed at preventing Down syndrome and other chromosomal aneuploidies through prenatal testing [2,10]. The successful execution of such disease prevention initiatives relies not only on the education and attitudes of professionals but also on the awareness and acceptance of the beneficiaries [6]. Therefore, this study aims to provide insights into the awareness of prenatal screening tests for genetic disorders among pregnant women in India and to examine how these factors correlate with demographic characteristics.

## Materials and methods

Study design

This hospital-based cross-sectional study was conducted in a tertiary care public hospital over a period of six months in 2023 by the Genetic Counselling Unit, Department of Anatomy, and Department of Obstetrics and Gynecology, All India Institute of Medical Sciences, Mangalagiri, India. Before the commencement of the study, ethical clearance was obtained from the Institutional Ethical Committee (AIIMS/MG/IEC/2022-23/240). The study was conducted among 200 pregnant women visiting the Department of Obstetrics and Gynecology for antenatal care. Since no similar studies were documented earlier in India, an assumption of awareness about prenatal screening tests among pregnant women to be 50% was made. Considering the power of the study as 80%, with a 95% confidence level and an absolute precision of 7%, the sample size was calculated as 196 and rounded off to 200. Pregnant women of all age groups willing to participate in the study were included. Pregnant women not willing to participate in the study or have already responded to the questions were excluded.

Data collection instrument

Data were collected using a self-administered questionnaire, both in English and the local regional language (Telugu). Translation into the local language was undertaken by a native language expert. The questionnaire contained two parts:

Part I: Participant information form to collect details regarding demographic characteristics like age of the mother, gestational age, parity, consanguinity, family history of genetic disease, educational status, and place of residence.

Part II: Awareness assessment form that includes eight questions to test awareness regarding prenatal screening tests among pregnant women. Questions were prepared based on the literature review and validated by subject experts [6-9]. Before the study was conducted, the questionnaire was pilot-tested among 20 pregnant women, who will not be a part of the study. Minor changes such as spelling, paraphrasing, and adding new words to clarify the questions were undertaken.

Statistical analysis

Awareness scores were generated from an aggregate of six questions. Each correct answer (yes) was scored as 1, while incorrect (no) and unanswered questions (do not know) received a score of 0. Thus, participants could achieve a maximum score of six and a minimum score of 0. Those who scored 4 or above were considered to possess adequate knowledge regarding prenatal screening tests, while those scoring 3 or below were categorized as having inadequate knowledge. The data obtained were tabulated and coded using a master sheet in Microsoft Excel. Statistical analysis was performed using GraphPad Prism software. Categorical variables were summarized as frequencies and proportions. The chi-square test was used to assess associations between demographic variables with awareness levels. Logistic regression analysis was done to identify factors associated with awareness. A p-value ≤0.05 was considered statistically significant.

## Results

A total of 200 pregnant women, attending the hospital for antenatal care, participated in the study, yielding a response rate of 100%. Table 1 presents the demographic characteristics of the 200 women who participated in the study.

**Table 1:** Socio-demographic characteristics of the participants

	Socio-demographic characteristics of the participants		n	%
1	Age of the pregnant women	<21 Yrs	40	20
		22-25 Yrs	72	36
		25-29 Yrs	69	34.5
		>30 Yrs	19	9.5
2.	Gestational age of fetus	First trimester	53	26.5
		Second trimester	61	30.5
		Third trimester	86	43
3	Parity	Primigravida	88	44
		Multigravida	112	56
4	Consanguinity	Consanguineous	26	13
		Non-consanguineous	174	87
5	Mode of conception	Natural conception	189	94.5
		Assisted conception	11	5.5
6	Family history of genetic disease	Yes	21	10.5
		No	179	89.5
7	Highest education	Degree	107	53.5
		High school	51	25.5
		Primary school	42	21
8	Employment	Salaried	26	13
		Self-employed	14	7
		Unemployed	160	80
9	Socioeconomic status	Upper class	03	1.5
		Middle class	169	84.5
		Below poverty line	28	14
10	Residence	Urban	71	35.5
		Semiurban	63	31.5
		Rural	66	33

**Figure 1:**
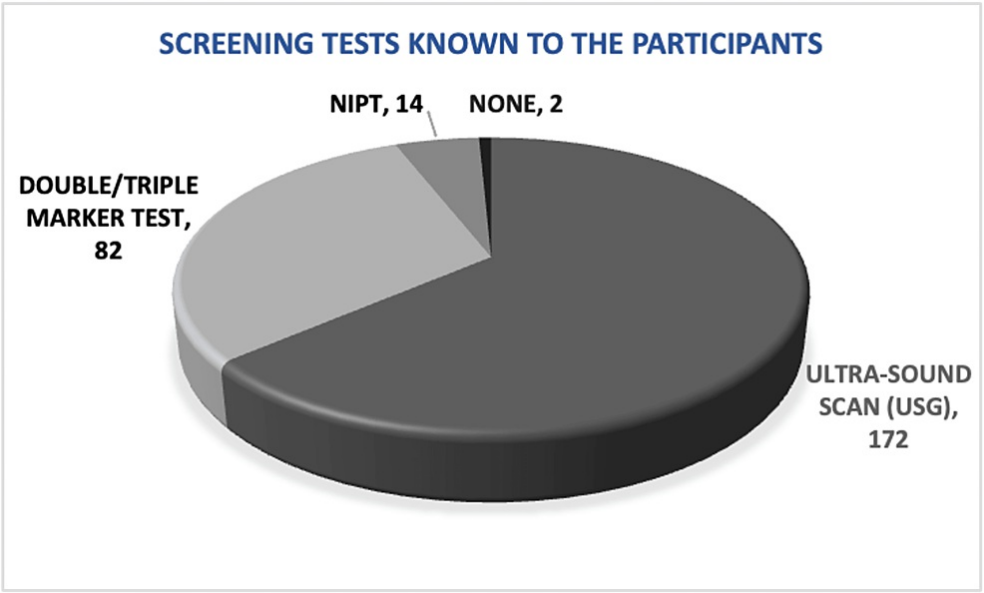
Pie chart showing the distribution of prenatal screening tests that the participants were aware of

**Figure 2:**
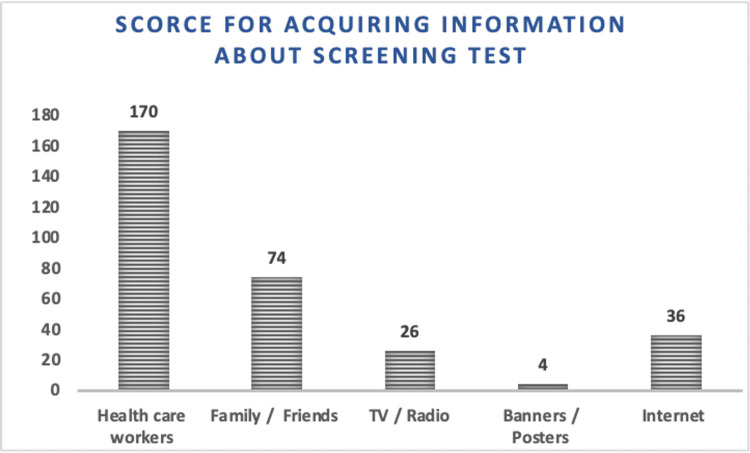
Bar diagram showing the sources from which the participants acquired information about prenatal screening tests

The majority of participants were familiar with ultrasound scans (172), while some were aware of the double/triple test (82). Only a small group (14) had knowledge about NIPT (Figure 1). They primarily relied on healthcare workers (170) for information about prenatal screening tests, followed by family/friends (74) and the internet (36) (Figure 2).

Nearly half of the participants were aware that prenatal screening tests can detect genetic disorders in the unborn baby (56%), can be performed starting from the 12th week of pregnancy onward (57%), and that the risk of having a baby with genetic disorders increases with the mother's age (51.5%). However, a smaller proportion of participants were aware that there is no risk of complications to the baby during prenatal screening tests (40%) and that a combination of ultrasound scans and blood tests can detect 80% of genetic disorders in the unborn child (43.5%). Notably, awareness about non-invasive prenatal testing (NIPT), was relatively low (7%) (Table 2). The awareness score, derived from the women's responses to the questionnaire, revealed adequate awareness among 73 (36.5%) participants and inadequate awareness among 127 (63.5%).

**Table 2:** Awareness levels of prenatal screening tests among pregnant women NIPT, non-invasive prenatal testing; USG, ultra-sound

	Awareness questionnaire	Yes	No	Do not know
		n (%)	n (%)	n (%)
1	Are you aware that prenatal screening tests can detect genetic disorders in the unborn baby?	112 (56)	50 (25)	38 (19)
2.	Are you aware that prenatal screening tests can be done starting from 12 weeks (3rd month) of pregnancy onward?	114 (57)	62 (31)	24 (12)
3	Are you aware that there is NO risk of complications to the baby while undergoing prenatal screening tests?	80 (40)	78 (39)	42 (21)
4	Are you aware that the risk of having baby a with genetic disorders increases with an increase in the mother’s age?	103 (51.5)	65 (32.5)	32 (16)
5	Are you aware that a combination of USG scan and blood tests can detect 80% of genetic disorders in the unborn child?	87 (43.5)	69 (34.5)	44 (22)
6	Are you aware of NIPT, which can be done using an unborn child’s DNA circulating in the mother's blood with an almost 99% detection rate?	14 (7)	116 (58)	60 (30)

Significant associations were observed between age groups and awareness levels (χ²=14.547, p=0.002). Specifically, women aged 22-25 years and 25-29 years exhibited higher levels of adequate awareness compared to those under 21 years old. Additionally, the gestational age of the fetus was found to be associated with awareness (χ²=7.908, p=0.019), with women in the third trimester demonstrating greater awareness compared to those in earlier trimesters. Furthermore, educational attainment showed a significant relationship with awareness (χ²=27.4322, p=0.001), where individuals with a bachelor's degree or higher exhibited higher levels of awareness compared to those with lower educational levels. Other socio-demographic factors such as parity, consanguinity, mode of conception, family history of genetic disease, employment status, socioeconomic status, and residence did not show significant associations with awareness levels (Table 3). 

**Table 3:** Association between demographic factors and awareness of prenatal screening tests among pregnant women

	Socio-demographic factor	Awareness of prenatal screening test				χ²	P-value
		Adequate		Inadequate			
		n	%	n	%		
1	Age of the pregnant women						
	<21 Yrs	10	13.7	30	23.6	14.547	0.002
	22-25 Yrs	18	24.6	54	42.5		
	25-29 Yrs	35	48.0	34	26.7		
	>30 Yrs	10	13.7	09	07.2		
2	Gestational age of the fetus						
	First trimester	11	15.0	42	33.0	7.908	0.019
	Second trimester	27	37.0	34	26.8		
	Third trimester	35	48.0	51	40.2		
3	Parity						
	Primigravida	34	46.5	54	42.5	0.382	0.536
	Multigravida	39	53.5	73	57.5		
4	Consanguinity						
	Consanguineous	12	16.5	14	11.0	1.201	0.272
	Non-consanguineous	61	83.5	113	89.0		
5	Mode of conception						
	Natural conception	67	92.0	122	96.0	1.635	0.200
	Assisted conception	06	08.0	05	04.0		
6	Family history of genetic disease						
	Yes	11	15.0	10	07.8	2.553	0.110
	No	62	85.0	117	92.2		
7	Highest education						
	Degree	56	76.7	51	40.2	27.4322	0.001
	High school	13	17.8	38	30.0		
	Primary school	04	05.5	38	29.8		
8	Employment						
	Salaried	14	19.1	12	09.4	4.4057	0.110
	Self-employed	06	08.2	08	06.3		
	Unemployed	53	72.8	107	84.3		
9	Socioeconomic status						
	Upper class	02	02.7	01	0.80	1.850	0.396
	Middle class	59	80.8	110	86.6		
	Below the poverty line	12	16.5	16	12.6		
10	Residence						
	Urban	24	32.8	47	37.0	0.8422	0.655
	Semiurban	22	30.1	41	32.3		
	Rural	27	37.1	39	30.7		
	Total	73	36.5	127	63.5		

In the logistic regression analysis examining factors associated with awareness regarding prenatal screening tests among pregnant women, several variables were considered. After adjusting for other variables, the age of the women, the trimester of the pregnancy, and the highest education level were found to be significant predictors of awareness. Specifically, women in the age group of 22-25 years (OR=2.321, p<0.05) and 25-29 years (OR=3.084, p<0.01) were significantly more likely to have adequate awareness compared to those aged less than 21 years. Additionally, women in the second trimester of pregnancy were more likely to have adequate awareness compared to those in the first trimester (OR=1.721, p<0.05). Furthermore, women with a bachelor's degree or higher education were more likely to have adequate awareness compared to those with lower levels of education (OR=2.156, p<0.001). 

## Discussion

The findings of this study shed light on the awareness of prenatal screening tests among pregnant women in our setting. The high response rate of 100% indicates strong engagement with the topic among participants. Our study revealed that 36.5% of the 200 participants had adequate knowledge regarding prenatal screening tests. Interestingly, our findings align with those from Turkey, where a study of 274 women reported a similar proportion (34.3%) with adequate knowledge [11]. In contrast, studies conducted in Nigeria reported lower percentages, with one study among 417 women indicating 18.5% and another among 422 women showing 17.1% [12,13]. Conversely, a study in Greece among 354 women found that 50.8% had adequate knowledge [8]. These variations across different populations underscore the influence of diverse socio-cultural factors, healthcare systems, and educational resources on the awareness levels of prenatal screening tests among pregnant women.

The awareness questionnaire highlighted varying levels of knowledge among pregnant women regarding prenatal screening tests. Overall, a substantial proportion of participants were aware of certain aspects, such as the ability of prenatal screening tests to detect genetic disorders in the unborn baby (56%) and the timing of when these tests can be conducted, starting from the 12th week of pregnancy onward (57%). However, a considerable number of respondents were unaware of the absence of risk to the baby during prenatal screening tests (40%) and the increased risk of genetic disorders with advancing maternal age (51.5%). Additionally, awareness regarding the effectiveness of a combination of ultrasound scans and blood tests in detecting genetic disorders was moderate (43.5%). 

At present, NIPT is accessible in more than 60 nations, including India [14]. Its application extends from screening for chromosomal abnormalities like trisomy 21, 18, and 13 to the screening of a broad spectrum of chromosomal anomalies across the genome [15,16]. Additionally, certain NIPT protocols incorporate screening panels for specific microdeletions [17,18]. In the present study, awareness about NIPT among pregnant women was notably low (7%). A study conducted in Saudi Arabia reported awareness levels of 43.9% among pregnant women, with a significant association between education level and knowledge of NIPT [19]. A Swedish study reported that 59.8% of expecting mothers were aware of NIPT [20]. A very high proportion (87.3%) of women in China were aware of NIPT, and this knowledge was associated with higher acceptability [21]. Education level appears to influence knowledge of NIPT, emphasizing the need for targeted educational interventions to enhance awareness.

Logistic regression analysis of the present series suggests that age, trimester of pregnancy, and education level are important determinants of awareness regarding prenatal screening tests among pregnant women. The significant associations between age groups and awareness levels suggest that younger pregnant women may require early educational interventions to improve awareness of prenatal screening tests. Similarly, the association between the trimester of pregnancy and awareness highlights the importance of timing in providing early information about these tests. Furthermore, the significant relationship between educational attainment and awareness emphasizes the role of education in shaping knowledge and understanding of prenatal screening tests. 

Limitations of the study

Despite the strengths of our study, several limitations should be acknowledged. First, the study was conducted in a single hospital setting, which may limit the generalizability of the findings to other populations. Additionally, the use of self-reported data may introduce bias, as participants may overestimate or underestimate their level of awareness. Furthermore, the cross-sectional design of the study precludes causal inference, and longitudinal studies are needed to explore changes in awareness over time.

## Conclusions

This study provides preliminary data on the awareness about prenatal screening tests among pregnant women in India since there is no such pre-existing information. It highlights the need for targeted interventions to improve awareness of prenatal screening tests among pregnant women, particularly focusing on younger age groups and those with lower levels of education. By addressing these disparities in awareness, healthcare providers can empower pregnant women to make informed decisions about their prenatal care, ultimately leading to improved maternal and child health outcomes.
